# The Potential Role of Connexins in the Pathogenesis of Atherosclerosis

**DOI:** 10.3390/ijms24032600

**Published:** 2023-01-30

**Authors:** Kajetan Kiełbowski, Estera Bakinowska, Andrzej Pawlik

**Affiliations:** Department of Physiology, Pomeranian Medical University, 70-111 Szczecin, Poland

**Keywords:** connexin, hemichannel, gap junctions, atherosclerosis, endothelial dysfunction, inflammation

## Abstract

Connexins (Cx) are members of a protein family which enable extracellular and intercellular communication through hemichannels and gap junctions (GJ), respectively. Cx take part in transporting important cell–cell messengers such as 3′,5′-cyclic adenosine monophosphate (cAMP), adenosine triphosphate (ATP), and inositol 1,4,5-trisphosphate (IP3), among others. Therefore, they play a significant role in regulating cell homeostasis, proliferation, and differentiation. Alterations in Cx distribution, degradation, and post-translational modifications have been correlated with cancers, as well as cardiovascular and neurological diseases. Depending on the isoform, Cx have been shown either to promote or suppress the development of atherosclerosis, a progressive inflammatory disease affecting large and medium-sized arteries. Cx might contribute to the progression of the disease by enhancing endothelial dysfunction, monocyte recruitment, vascular smooth muscle cell (VSMC) activation, or by inhibiting VSMC autophagy. Inhibition or modulation of the expression of specific isoforms could suppress atherosclerotic plaque formation and diminish pro-inflammatory conditions. A better understanding of the complexity of atherosclerosis pathophysiology linked with Cx could result in developing novel therapeutic strategies. This review aims to present the role of Cx in the pathogenesis of atherosclerosis and discusses whether they can become novel therapeutic targets.

## 1. Introduction

Cardiovascular diseases (CVD) represent the leading cause of death globally. Atherosclerosis is a major risk factor for CVD and takes part in the development of coronary artery disease, cerebrovascular events, and peripheral artery disease. The global burden of CVD is significant, as up to 42% of the general population develops subclinical coronary atherosclerosis [1], a progressive inflammatory disease that affects large and medium-sized arteries. Local fat deposition in the vascular wall is a hallmark of atherosclerosis, and advanced disease is characterised by extensive plaque formation. Atherosclerotic lesions narrow a vessel and may lead to an organ infarct, while a plaque rupture can create a blood clot [2]. One of the key structures in the pathogenesis of atherosclerosis is the endothelium. It separates the wall of the blood vessel from the lumen, and its dysfunction, followed by the accumulation of low-density lipoprotein (LDL), is the main inducing process of the disease [3,4]. It has been found that endothelial cells express communicating molecules known as connexins (Cx), such as Cx37, Cx40, and Cx43 [5]. Expression of these structures has also been identified in macrophages [6], one of the key cells in atherosclerosis, which develop into foam cells. The aim of this review is to present the role of connexins in the pathogenesis of atherosclerosis and discuss whether they can become novel therapeutic targets.

## 2. Connexins: Structure and Biology

Connexins belong to a protein family that forms single transmembrane channels known as hemichannels and more complex gap junctions (GJ), which take part in intercellular transportation. The family consists of 21 proteins, and they are expressed in every human body system. Cx43 is one of the best-known members, and its expression has been found in approximately 100 human cell types [7]. Cx have been further grouped into five subfamilies: GJA, GJB, GJC, GJD, and GJE, according to the similarities in their gene sequences [8].

**Figure 1:**
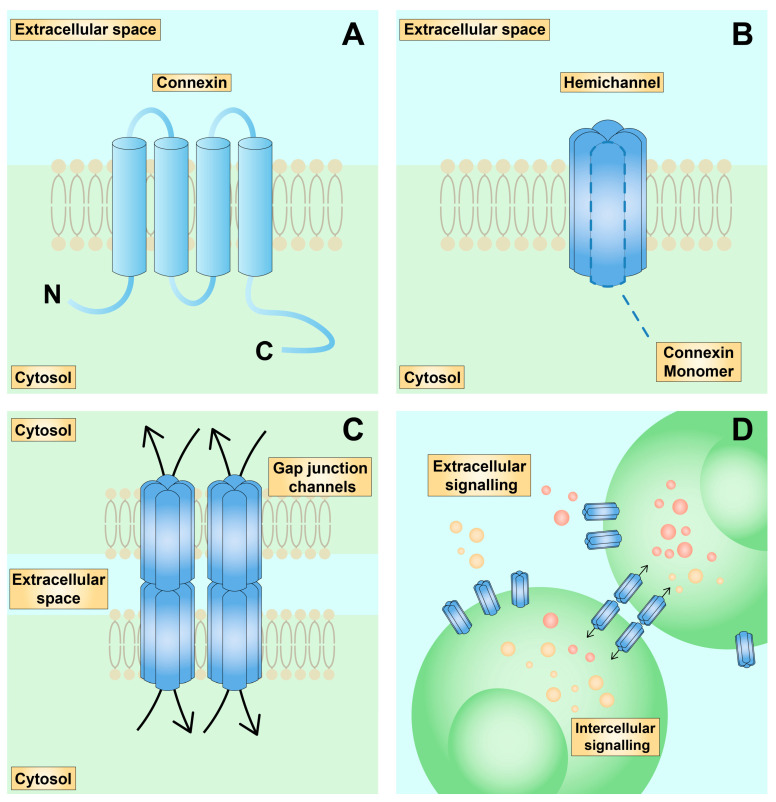
Structure of channels formed by connexins. (**A**): Schematic representation of a connexin composed of four transmembrane domains, two extracellular loops, one cytoplasmic loop, and carboxyl- and amino-termini. (**B**): Schematic representation of a hemichannel which consists of six subunits. (**C**): Schematic representation of gap junctions formed by two hemichannels. (**D**): Schematic representation of inter- and extracellular communication through gap junctions and hemichannels, respectively.

The structure of Cx consists of carboxyl- (CT) and amino-termini (NT), four transmembrane domains, two extracellular loops, and a cytoplasmic loop (CL; Figure 1A). Despite being highly conserved molecules, variations occur in the loops and CT regions [9]. CT and its post-translational modifications play a significant role in Cx biology by regulating their ubiquitination and assembly into more advanced structures [10,11].

Cx are grouped into hexamers and form a hemichannel (Figure 1B). Hemichannels from opposing cells together create a GJ (Figure 1C). Hemichannels allow for communication between cells and the extracellular environment, while GJ enable intercellular molecule transport (Figure 1D) [12]. CL and CT act as regulators of hemichannel and GJ activity [13]. In addition, extracellular loops of Cx containing cysteine control the assembly and activity of hemichannels and GJ [14,15]. Various molecules are transported through Cx, including 3′,5′-cyclic adenosine monophosphate (cAMP), inositol 1,4,5-trisphosphate (IP3), Ca^2+^, and ATP, which are important cell–cell messengers [16,17]. However, the permeability of these channels is selective according to the isoform of the molecule. Additionally, the activity of Cx channels changes depending on the environment. Pro-inflammatory stimulation by tumour necrosis factor α (TNF-α) and interleukin-1β (IL-1β) regulate the permeability of hemichannels [18]. IL-1β, together with tumour growth factor-β (TGF-β), were found to increase the expression of Cx43 in cardiac fibroblasts [19] and cardiomyocytes [20]. Furthermore, Cx expression is further regulated by small non-coding RNA molecules known as microRNA (miRNA), such as miR-206, miR-155, and miR-144-3p [21,22,23]. In addition, Cx do not only form channels in the cell membrane, but also in the mitochondria [24]. Recent studies show that Cx-based communication is essential in regulating cellular functions, such as proliferation or differentiation [25,26,27,28]. Alterations in Cx distribution, degradation, and post-translational modifications have been linked with the pathophysiology of various diseases of the heart (atrial fibrillation [29,30]), brain (Alzheimer disease [31]), and skin (eczema) [32], among others. Moreover, Cx play a role in cancer progression and the development of metastasis [33]. To this date, approximately 900 mutations in GJ genes have been identified, associated with over 30 different diseases [34].

## 3. Connexins in Endothelial Dysfunction

**Figure 2:**
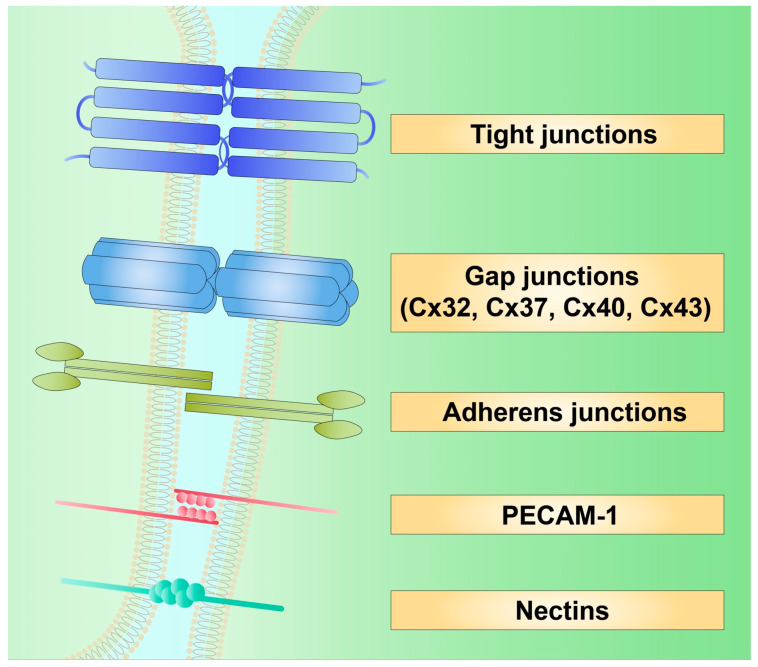
Cell–cell junctions between endothelial cells. Cx—connexin; PECAM-1—platelet endothelial cell adhesion molecule 1.

The development of atherosclerosis comprises several steps. In short, these include endothelial dysfunction, LDL infiltration, foam cell formation, and development of a fibrous plaque [35]. Endothelial cells (EC) create a monolayer separating the vessel from the lumen and take part in maintaining fluid homeostasis. EC prevent platelet activation and thrombus formation [36,37,38], and regulate vasodilation and vasoconstriction by producing nitric oxide (NO) through endothelial NO synthase (eNOS) [39] and endothelin, respectively [40]. EC further regulate blood pressure as they express prostaglandin receptors and angiotensin convertase enzyme (ACE), which transforms angiotensin I (Ang I) into angiotensin II (Ang II). The prostaglandin E2 receptor EP4 has been found to decrease blood pressure, while the effects of Ang II depend on the type of receptor it stimulates [41]. The activation of angiotensin type 1 receptor (AT1R) causes vasoconstriction and is associated with pro-inflammatory conditions [42]. On the contrary, the stimulation of type 2 receptor (AT2R) is correlated with vasodilation and increased production of NO [43]. EC are tightly coupled with surrounding cells through several cell–cell junctions. These include channels and adherent proteins that are used for the transport of fluids, ions, and other molecules. They form a barrier that blocks the diffusion of macromolecules through EC. Furthermore, they create conditions for the electrical potential of the endothelium to be conducted. Those coupling structures include tight junctions, gap junctions, adherens junctions, nectins, and platelet endothelial cell adhesion molecule 1 (PECAM-1; Figure 2) [44,45]. Cx are expressed in the endothelium of different vascular beds and include Cx32, Cx37, Cx40, and Cx43 [46]. Cx43 has been found to also take part in vasculogenesis, as it forms GJ between pericytes and EC precursors [47].

**Figure 3:**
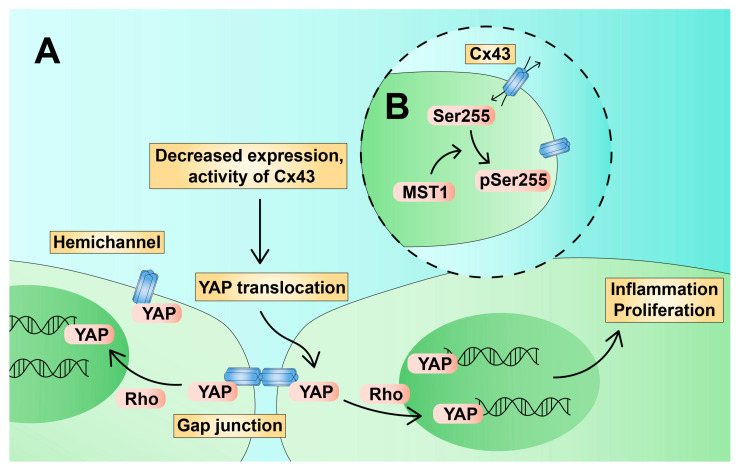
Hypothetical impact of decreased expression or activity of Cx43 on YAP translocation and induction of a pro-inflammatory state of the endothelium. (**A**): Quiescent endothelium with the YAP protein anchored on Cx43. Cx43 downregulation promotes YAP translocation into the nucleus. (**B**): Inhibition of Cx43 via Ser255 phosphorylation by MST1. Cx43—connexin 43; YAP—Yes-associated protein.

Endothelial dysfunction (ED) is a progressive disorder related to decreased NO production, increased secretion and sensitivity to vasoconstrictors, and shear stress. In addition, one of the hallmarks of ED is increased production of reactive oxygen species (ROS). ED is associated with changes in vascular tone, endothelial permeability, and leukocyte diapedesis. Vascular stiffness is one of the processes that has been linked with endothelial inflammation and the development of atherosclerosis [48]. Okamoto et al., in a series of experiments on human umbilical vein endothelial cells (HUVEC) and human aortic and lung EC, have found that the blockage of Cx32 and Cx43 GJ increases endothelial cellular stiffness [49]. It is associated with monocyte adhesion, and thrombomodulin may soften the endothelium by promoting GJ communication [50]. Furthermore, an in vitro (HUVEC) and in vivo (mouse) study revealed that the inhibition of Cx32 enhances the expression of tissue factor (TF), which stimulates coagulation process and is present in atherosclerotic plaques in high levels [51,52]. Therefore, Cx32 could be considered protective channels. Several factors act synergistically to shift EC into a pro-inflammatory state, including ageing, obesity, dyslipidaemia, smoking, hyperglycaemia, and microbiome alterations, among others [53]. Elevated ROS and dyslipidaemia cause the formation of oxidised LDL (ox-LDL), which has been found to take part in ED [54,55]. Hu et al. demonstrated that ox-LDL induces the overexpression of miR-496, which results in increased apoptosis and dysfunction of endothelial cells. In addition, miR-496 decreases the expression of Yes-associated protein 1 (YAP1), which is an effector of the Hippo pathway. The Hippo–YAP axis takes part in endothelial proliferation and vascular remodelling [56]. Furthermore, Hatabi et al. have found that YAP is colocalised with Cx43 in the cell membrane of muse cells. Muse cells are pluripotent cells capable of differentiating into numerous cell subtypes, including endothelium. YAP is a transcription factor that is anchored within GJ. The inhibition of GJ leads to YAP translocation into the nucleus and subsequent upregulation of selected genes associated with pluripotency [57]. Furthermore, inhibition of Cx43 hemichannels was found to increase YAP nuclear translocation in astrocytes [58]. Moreover, changes in YAP activity have been linked with blood flow alterations. Disturbed flow results in the translocation of YAP into the nucleus and its activation, which shifts EC into a pro-inflammatory and proliferative state, which is typical for atherosclerosis [59,60,61]. In addition to shear stress, Jia et al. showed that TNF-α, a pro-inflammatory cytokine, also induced YAP translocation and the transcription of adhesion molecules [62]. The YAP transcriptional function is activated through the Rho family of GTPases. YAP is involved in the regulation of several inflammatory and fibrotic genes, including *IL1RAP*, *IL6ST*, *FAS*, *CASP4*, *CD44*, *EGR1*, *ICAM1*, *TGFB1*, *MMP16*, *ADAM9*, and *LOXL2*, among others [63]. Accordingly, TNF-α stimulation was found to decrease the expression of Cx43 in chondrocytes, which could explain YAP translocation (Figure 3) [64]. In contrast, TNF-α stimulation, together with non-uniform shear stress, upregulates Cx43 in HUVECs [65]. According to an older study by van Rijen et al., TNF-α downregulates Cx40 and Cx37 but does not impact mRNA expression of Cx43 in HUVEC. However, TNF-α redistributes Cx43 and reduces GJ-mediated dye coupling [66]. In addition, Cx43 interacts with another member of the Hippo pathway, mammalian sterile 20-like kinase 1 (MST1). MST1 phosphorylates Cx43 at Ser255, which inhibits hemichannel activity but does not impair intercellular junctions. Hemichannel inhibition has been correlated with suppressed disturbed flow-induced endothelial activation and dysfunction. Moreover, inhibited activation of endothelium and atherosclerosis by MST1 was observed in an in vivo study [67].

TNF-α is one of the cytokines released from EC after stimulation with ox-LDL. Modified lipoprotein uptake is performed via lectin-like oxidized low-density lipoprotein receptor 1 (LOX-1). The subsequent activation of nuclear factor kappa B (NF-κB) induces the secretion of other pro-inflammatory molecules such as interleukin 6 (IL-6) and monocyte chemoattractant protein-1 (MCP-1) [68,69,70]. In addition, NF-κB activation increases the production of ROS and the activity of NADPH oxidase [71]. Nevertheless, the impact of ox-LDL on the expression of Cx seems to be more complex. Liu et al. and Zhang et al. showed that Cx43 expression in HUVEC increased after treatment with ox-LDL [72,73]. Additionally, Alonso et al. demonstrated that increased Cx43 could be the result of NF-κB activation by Ang II in murine aortas [74]. Previous reports also demonstrated that NF-κB is a downstream of LOX-1 in endothelial cells [75]. Therefore, these findings suggest that NF-κB could contribute to the upregulation of Cx43, but this issue should be further examined. In addition, the role of ox-LDL-stimulated transcription factors in the expression of Cx43 requires more research. Activator protein 1 (AP-1) is another transcription factor that could link ox-LDL and Cx43 [76,77,78,79].

**Figure 4:**
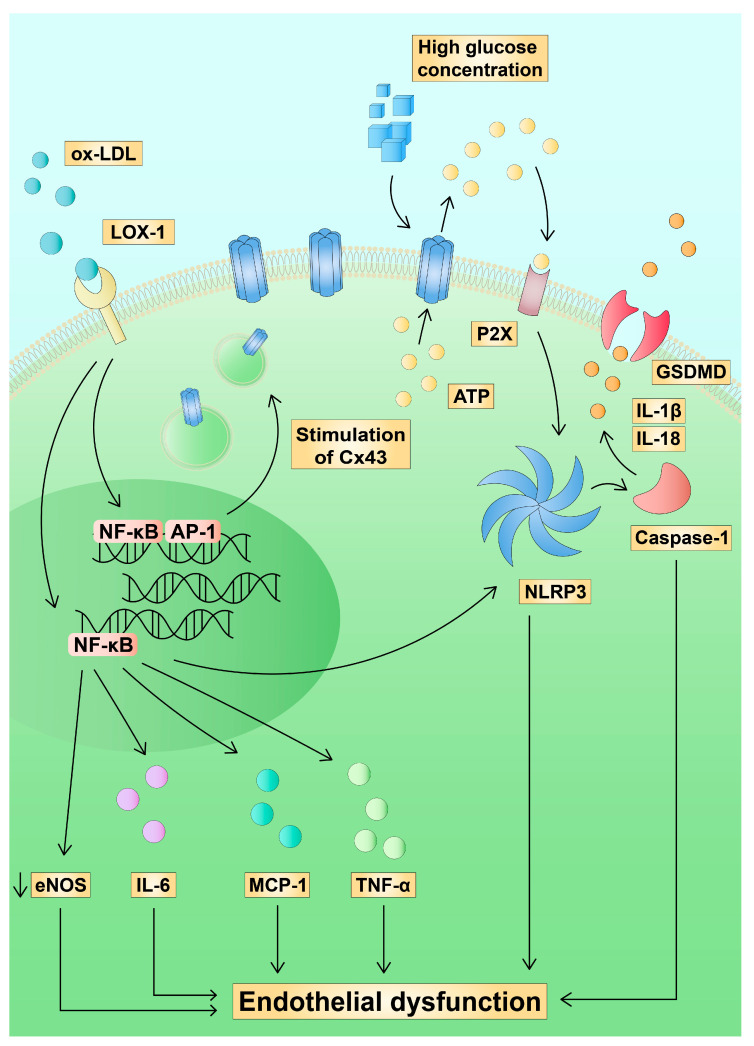
Hypothetical impact of Cx43 on endothelial dysfunction. Ox-LDL-induced activation of NF-κB leads to Cx43 upregulation. Ox-LDL and high glucose concentration contribute to Cx43 opening and ATP release which stimulate P2X purinergic receptors and activate the NLRP3 inflammasome. Subsequently, caspase-1 promotes pro-inflammatory IL-1β and IL-18. AP-1—activator protein 1; ATP—adenosine triphosphate; Cx43—connexin 43; eNOS—endothelial NO synthase; GSDMD—gasdermin D; IL-1β—interleukin 1β; IL-18—interleukin 18; IL-6—interleukin 6; LOX-1—lectin-like oxidized low-density lipoprotein receptor 1; MCP-1—monocyte chemoattractant protein-1; NF-κB—nuclear factor kappa B; NLRP3—nod-like receptor protein 3 inflammasome; ox-LDL—oxidised LDL; P2X—purinergic receptor; TNF-α—tumour necrosis factor alpha.

Moreover, ox-LDL has been found to suppress cholesterol efflux from EC and, together with NF-κB, to induce nod-like receptor protein 3 (NLRP3) inflammasome assembly [80,81]. Furthermore, smoking and hypertension, significant risk factors for atherosclerosis, have been also identified as NLRP3 activators [82,83]. NLRP3 is a pattern recognition receptor (PRR) and is activated by pathogen-associated molecular patterns (PAMP) and damage-associated molecular patterns (DAMP). NLRP3 assembly promotes the secretion of interleukin-1β (IL-1β) and interleukin-18 (IL-18) through caspase-1 activation. The inflammasome is also involved in a cell death process known as pyroptosis through the stimulation of gasdermin D (GSDMD), which creates pores in the cell membrane. NLRP3 plays a role in the pathophysiology of numerous diseases, including Parkinson’s disease [84], diabetes [85], inflammatory bowel disease [86], COVID-19 infection [87], and atherosclerosis [88], among others. Zheng et al. have revealed that NLRP3 is overexpressed in the aorta of patients with coronary atherosclerosis [89]. Recent evidence suggests that NLRP3 assembly is linked with the activity of Cx. The inflammasome can also be activated by ATP through purinergic receptors, such as P2X7 (Figure 4) [90,91,92]. High glucose concentrations have been found to induce hemichannel opening and release of ATP, which contributes to ED. Simultaneously, high glucose levels and interferon-γ (IFN-γ) cause a reduction in cell–cell GJ communication. [93]. In addition, Lyon et al. have found that high glucose concentrations and inflammatory cytokines in human retinal epithelial cells increase ATP release from Cx43 and activate NLRP3 assembly [94]. Since NF-κB could upregulate Cx expression, this transcription factor could also indirectly contribute to the subsequent ATP release from hemichannels. NF-κB and Cx43 may be connected through cyclooxygenase 2 and prostaglandin E_2_, which increase the cytoplasmic levels of Ca^2+^ by interacting with EP_1_ receptors. The impact of elevated intracellular Ca^2+^ on hemichannel activity has been found in several cell types [95,96,97]. Furthermore, GJs are permeable to Ca^2+^ and these structures take part in intercellular signalling [98]. It has been also found that blocking P2X7 receptors attenuates inflammatory changes induced by ox-LDL [99,100]. Furthermore, Jin et al. revealed that ox-LDL contributes to ATP release into the extracellular medium in human endothelial cells [101].

Other crucial steps in ED are cell adhesion and leukocyte diapedesis. Due to the endothelial activation by cardiovascular risk factors, expression of cell adhesion molecules (CAM) is increased. These molecules include vascular cell adhesion molecule 1 (VCAM-1), intercellular adhesion molecule 1 (ICAM-1), E-selectin, and P-selectin. CAM facilitate monocyte recruitment and migration to the vessel intima [102]. Various proteins or genes stimulate the expression of CAM and promote atherosclerosis, including histone deacetylases 1 and 2 (HDAC1/2) [103], epithelial–stromal interaction 1 gene (EPSTI1) [104], YAP [105], and NF-κB [106], among others. Several reports have linked the overexpression of Cx and CAM (ICAM-1, VCAM-1) [107,108,109]. The mitogen-activated protein kinase (MAPK) and phosphoinositide 3-kinase/protein kinase (PI3K) pathways have been found to connect Cx and CAM [107,110].

**Figure 5:**
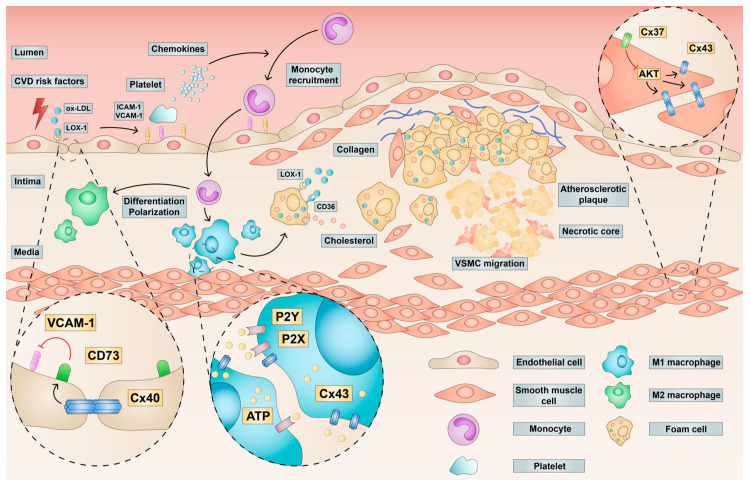
Development of an atherosclerotic plaque and hypothetical roles of Cx in this process. Cx40 stimulates expression of CD73 which has antiadhesive properties. ATP release through Cx43 contributes to macrophage activation and M1 polarisation. Cx37 suppresses AKT protein and disrupts the Cx43. ATP—adenosine triphosphate; Cx—connexin; ICAM-1—intercellular adhesion molecule 1; LOX-1—lectin-like oxidized low-density lipoprotein receptor 1; ox-LDL—oxidised LDL; P2X/P2Y—purinergic receptors; VCAM-1—vascular cell adhesion molecule 1; VSMC—vascular smooth muscle cell.

Taken together, the role of Cx in ED is complex. Blocking Cx could contribute to increased endothelial stiffness and to the translocation of YAP protein to the nucleus. On the other hand, overexpression of Cx is associated with NF-κB activation and the stimulation of CAM. Hyperglycaemia and ox-LDL, important risk factors for atherosclerosis, might induce ATP release through hemichannels, which further stimulates the secretion of pro-inflammatory cytokines. Therefore, current evidence suggests that Cx43 has proatherogenic activity. Additionally, Shi et al. found that the inflammatory response (TNF-α, IL-1β, ICAM-1), after high glucose stimulation of human endothelial retinal cells, was increased after Cx43 overexpression. Cx43 knockdown in mouse models partially inhibited these effects [108]. This finding was further supported in another study by Ramadan et al., who demonstrated that ionising radiation, another risk factor for the development of atherosclerosis, increases the expression of Cx43 and downregulates Cx40 in the endothelium [111]. Cx40 was found to have the opposite effects of Cx43. For instance, Cx40 can silence NF-κB and, therefore, promote a quiescent endothelium [112]. In addition, Cx40 enhances endothelial CD73 activity in mice models, which has antiadhesive properties (Figure 5). Cx40 inhibition results in a decrease in CD73, which stimulates leukocyte adhesion. The activity of CD73 results in the generation of adenosine, which in turn stimulates A_2_B receptors. The enhancement of A_2_B signalling promotes GJ-mediated communication [113]. Moreover, Grünewald et al. demonstrated that CD73-deficient HUVECs undergo a pro-inflammatory morphological change, become more permeable, and increase CAM expression [114]. In addition, the expression of Cx40 is further regulated by the short isoform of the Tet methylcytosine dioxygenase 1 (TET1s) through histone acetylation. Cx40 is required for an endothelial barrier enhancement stimulated by TET1s [115]. Furthermore, Denis et al., in the series of experiments on mouse and human ECs, revealed that Cx40 expression is regulated by shear stress through Krüppel-like transcription factor 4 (KLF4) [116]. KLFs take part in regulating endothelial homeostasis and Cx expression. KLF2 has been found to induce the expression of endothelial Cx37 and to promote Cx37 GJ communication under laminar shear stress in bEnd.3 endothelial cells, and was also linked with a quiescent endothelium [117]. Nevertheless, there is a knowledge gap in the association between Cx37 and ED.

Therefore, the disproportional activity of hemichannels has been associated with ED. Pro-inflammatory and atherogenic factors stimulate the expression of Cx43. MST1 phosphorylates Cx43 at Ser255, which inhibits the activity of hemichannels. However, oscillatory shear stress reduces the expression of MST1, and subsequently promotes the opening of membrane-bound channels. Furthermore, ATP release through hemichannels may result in NLRP3 assembly and further secretion of pro-inflammatory cytokines.

Additionally, the dysregulation of GJ intercellular communication has been found to promote pro-inflammatory conditions in endothelium. To begin with, the blocking of intercellular communication may contribute to the vascular stiffness. Secondly, direct intercellular communication between TNF-α stimulated and non-stimulated EC promotes EC activation and secretion of tissue factor. The redistribution of GJ may be closely correlated with the activity of the YAP transcription factor. Furthermore, disturbed flow changes the distribution of GJ in favour of Cx43, together with a reduction in membrane eNOS [118]. Endothelial NOS is located closely to the Cx40 and Cx37, and the latter connexin was found to directly interact with the reductase domain of eNOS and regulate its activity [119]. However, Alonso et al. showed that NO secretion is disturbed in aortas of Cx40-/- mice. Cx40 and Cx37 are linked and alterations in Cx40 expression can impact Cx37 [119,120].

## 4. Connexins in the Activation of Platelets and Macrophages

Under physiological conditions, platelets do not adhere to the endothelium. However, after EC shift into a pro-inflammatory state that stimulates the expression of CAM, platelets begin to bind to the endothelium. Subsequently, platelets change their shape and secrete numerous chemokines that attract and activate other cells. Cx play a role in platelet activation, and previous studies have revealed the presence of Cx40 [121], Cx37 [122], and Cx62 [123] on these cell fragments. Similar to EC, platelets express purinergic receptors (P2X), which stimulate platelet activation [124]. Furthermore, platelet ATP release has been found through another transmembrane protein, pannexin 1. Furthermore, release is necessary to support thrombin formation and platelet aggregation [125]. Following activation, platelet–platelet, platelet–endothelium, and platelet–leukocyte interactions are induced. CD40L is an important molecule expressed on platelets and is responsible for leukocyte recruitment through MCP-1, also known as CCL2 [126]. CCL4, CCL5, CXCL4, CXCL5, CXCL12, and CXCL16 represent other chemokines secreted by activated platelets [127].

Due to the expression of CAM on the surface of the endothelium and the secretion of chemokines by platelets, the next step in the development of atherosclerosis is monocyte recruitment. The main steps of this process include rolling, adhesion, activation, and transmigration [35]. Cx located on monocytes were found to regulate the recruitment process. Wong et al. showed that Cx37 prevents monocyte recruitment, while its knockdown enhances the adhesion process in an in vitro study and in an animal model [128]. On the other hand, Cx43 was found to stimulate leukocyte adhesion. Ji et al. demonstrated that propofol decreased Cx43 expression on monocytes, which inhibited cell adhesion [129]. In the vessel intima, monocytes differentiate into macrophages. Depending on the microenvironment, macrophages polarise into several subtypes. M1, M4, and Mox are pro-inflammatory cells that secrete cytokines such as IL-1β, IL-6, IL-12, and TNF-α. M2, M(Hb), and Mhem represent anti-inflammatory macrophages that produce IL-4 and IL-10, cytokines that prevent plaque formation [130]. The Cx43/NF-κB axis stimulates M1 polarisation, and blockage of this connexin inhibits the expression of M1-related proteins [131,132]. The distribution of these subtypes is different throughout atherosclerotic plaques. M1 dominance is present in rupture-prone and symptomatic plaques, while an increased number of M2 cells is found in more stable regions [133,134]. Inside the vessel wall, macrophages internalise LDL and ox-LDL through scavenger receptors, LOX-1, SR-A1, and CD36 [135]. Ox-LDL stimulation leads to NF-κB and NLRP3 activation, which subsequently induces the further secretion of inflammatory cytokines, such as IL-1β, IL-6, IL-18, and TNF-α [136,137,138]. Furthermore, ox-LDL reduces the expression of Cx37 and upregulates Cx43 [139]. These alterations further promote monocyte recruitment and ED. Nevertheless, the precise role of Cx in this process is not fully understood. Again, as in EC and platelets, it is suggested that ATP release might take place and be responsible for inflammatory events downstream.

To begin with, Stachon et al. have found that ATP promotes atherosclerosis by inducing leukocyte adhesion [140]. Several other studies confirmed that the depletion of various purinergic receptors is associated with reduced inflammation and limited plaque formation [141,142,143]. Intriguingly, in an animal model and in vitro experiments, Dosch et al. found that the ATP release through Cx43 promotes the M1 macrophage polarisation after the stimulation of toll-like receptors (TLRs) [144]. Furthermore, Choi et al. in in vitro and in vivo experiments demonstrated that Cx43 knockout inhibited ATP release from macrophages and suppressed inflammasome assembly [145]. In addition, depending on the subtype of the macrophage, these cells express various purinergic receptors. For instance, A2A, P2Y13, P2Y14, and temporarily P2X7 (responsible for NLRP3 activation) are typically upregulated in M1, while P2Y1 and P2Y6 are exclusively expressed by M2 cells [146]. Therefore, Cx43 upregulation and ATP release might have different outcomes depending on the microenvironment in the atherosclerotic plaques. For instance, ATP released through hemichannels could contribute to M1 polarisation and NLRP3 assembly, which, in turn, further stimulates the secretion of pro-inflammatory cytokines and monocyte recruitment. The uptake of modified LDL and cholesterol leads to foam cell formation (Figure 5). Subsequently, foam cells accumulate, which contributes to the growth of atherosclerotic plaques.

Overall, a limited number of studies have confirmed the presence of hemichannels on platelets and functional GJ formation between them. Therefore, future studies should focus on examining the role of Cx in physiology and pathology of platelets. In macrophages, ATP release through hemichannels might contribute to M1 polarisation and pro-inflammatory activation. Under non-inflammatory conditions, macrophages do not form GJ. However, these cells may form homo- or heterocellular functional GJ under stimulation with certain pro-inflammatory molecules. GJ-mediated communication takes part in inflammatory responses, such as the secretion of metalloproteinase MMP-2 [147].

## 5. Connexins and Vascular Smooth Muscle Cells

Vascular smooth muscle cells (VSMC) play a significant role in blood flow homeostasis due to their contractile properties. Nevertheless, under stressful conditions, VSMC significantly contribute to the development of atherosclerotic plaques. For instance, they increase the foam cell population. Approximately 50% of coronary atherosclerotic foam cells are VSMC-derived [148]. In addition, VSMC take part in calcium deposition and in the formation of the fibrous cap that covers the plaque, preventing its rupture. High concentrations of modified lipoproteins also stimulate VSMC to further secrete chemokines, thereby contributing to monocyte recruitment [35]. These numerous roles are possible due to the dynamic feature of phenotype switching. VSMC subtypes include macrophage-like cells, foam cells, osteochondrogenic cells, and myofibroblast-like cells, among others [149]. VSMC are activated by various stimuli, including platelet-derived growth factor (PDGF), insulin-like growth factor (IGF-1), epidermal growth factor (EGF), and transforming growth factor-β (TGF-β). Subsequently, VSMC proliferate, migrate, and deposit collagen [150]. Cx37 has been found to suppress VSMC proliferation. In addition, the expression of Cx37 decreases the activity of serine/threonine protein kinase AKT, which phosphorylates Cx43 and promotes GJ communication [151,152]. Therefore, Cx37 has the opposite effects of Cx43 in the vasculature, but also causes its destabilisation. On the contrary, ox-LDL induces Cx43 overexpression and phenotype switching into a synthetic subtype. The blockage of Cx43 GJ suppresses VSMC migration and proliferation in rat aortic VSMC [153].

One of the most significant processes used to remove lipid droplets from VSMC is autophagy. It involves encapsulating the substrates into autophagosomes and their subsequent merging with lysosomes, exposing the contents to proteases and hydrolases [154]. It has been debated whether autophagy plays a cytoprotective or cytotoxic role, but it is now considered that autophagy is an atheroprotective process. Nevertheless, impaired autophagy in VSMC leads to a process known as senescence, in which VSMC do not grow but remain metabolically active [155]. Telomere damage is one of the mechanisms correlated with cell senescence. These cells form micronuclei and induce pro-inflammatory signalling pathways by producing IL-1α, IL-1β, IL-8, CCL2, and CCL20 [156]. In atherosclerosis, senescent VSMC inhibit the generation of the fibrous cap, which increases the risk of plaque rupture [157]. Several other studies have also concluded that VSMC senescence promotes atherosclerosis [158,159,160]. Robichaud et al. showed that autophagy is largely defective in lipid-loaded VSMC [161]. Furthermore, Pi et al. demonstrated that the purinergic receptor P2Y12 enhances VSMC-derived foam cell formation by suppressing autophagy [162]. P2Y12 is a G-protein-coupled receptor activated by adenosine diphosphate (ADP). However, VSMC perform extracellular ATP hydrolysis, which generates ADP [163]. Moreover, Qin et al. have shown that Cx43 takes part in an ox-LDL-induced inhibition of VSMC autophagy through the PI3K pathway in cells obtained from the thoracic aortas of rats. The authors showed that suppressing Cx43 improves this process and attenuates foam cell formation [164]. Therefore, activated VSMC could secrete ATP through upregulated Cx43 hemichannels. Subsequently, ATP would be hydrolysed into ADP, which may interact with the P2Y12 receptor and suppress protective autophagy.

Taken together, VSMC significantly contribute to the development of atherosclerosis. Recent evidence suggests that impaired hemichannel and GJ may regulate the pro-inflammatory responses. As in EC, platelets, and macrophages, ATP release might represent the potential role of hemichannels in the pathogenesis of atherosclerosis. GJ-mediated intercellular communication plays a role in VSMC contraction. Deng et al. demonstrated that Cx43 GJ are necessary for Ang II to induce a cell contraction. The inhibition of intercellular junctions leads to vascular dilation [165]. Connections between EC and VSMC are known as myoendothelial gap junctions (MEJ). Zhang et al., in studying human coronary artery endothelial and smooth muscle cells (HCAEC, HCASMC), found that MEJ take part in SMC phenotype switching. The authors suggested that the exposure to the shear stress may disrupt the distribution of MEJ subtypes from heterotypic Cx43/Cx40to homotypic Cx43/Cx43 junctions, which subsequently promotes synthetic phenotypes of SMC [166]. The summary of different roles of Cx isoforms in the promotion or suppression of atherosclerosis is shown in Table 1.

## 6. Connexin Inhibitors

Since Cx have been linked with the pathophysiology of numerous diseases in various human systems, these proteins have long been considered therapeutic targets. Cx43 is the most studied connexin and, as demonstrated in this review, its overexpression is strongly associated with the development of atherosclerosis. Cx inhibitors include mimetic peptides, miRNAs, short-interfering RNAs (siRNAs), and chemical-based agents. In addition, alterations in extracellular calcium have also been associated with the opening and closing of hemichannels [167,168].

Mimetic peptides (MP) contain short sequences of Cx and were designed to modulate GJ. On the contrary, they have been found to bind to unpaired hemichannels and inhibit their function. At the same time, Cx-MP are considered not to alter already existing junctions [169]. However, since MP inhibit the docking of hemichannels and the formation of new intercellular junctions, GJ activity should decrease in a time-dependent manner. GAP26 is a synthetic MP of the first Cx43 extracellular loop. It has been demonstrated in cellular and animal models that the use of GAP26 can protect against cardiac ischemia-reperfusion injury [170,171], bronchopleural dysplasia [172], and atherosclerosis [164]. Other MP-targeting Cx43 are GAP27 (second extracellular loop) [173], GAP19 (impaired interaction between CT and CL) [174], Peptide5 (second extracellular loop) [175], SRPTEKT-Hdc [176], and αCT1 (carboxyl-terminus) [177]. The use of GAP26 and GAP27 has been examined in various cells and tissues, including endothelium, lymphocytes, heart, and astrocytes, among others [178]. The topical use of αCT1 to improve wound healing has been evaluated in a clinical trial by Grek et al. The adverse effects (AE) of αCT1 were mild and rates were not significantly different from the control group The authors observed improvements in wound healing in the study group [179]. Furthermore, Ghatnekar et al. in a randomised clinical trial assessed the efficacy and safety of αCT1 in patients with venous leg ulcers. A greater reduction of the ulcer area was observed in the MP group, while AE did not differ significantly between control and study groups [180]. The efficacy and safety of αCT1 was also noted in the treatment of diabetic foot ulcers [181]. Moreover, JM2, another MP containing the C-terminus sequence of Cx43, was found to significantly suppress ATP release through these connexins [182]. Nevertheless, the efficacy and safety of the systemic use of mimetic peptides applied for the treatment of cardiological diseases remain unknown due to the lack of clinical trials.

SiRNAs are based on the RNA interference process, which involves the RNA-induced silencing complex (RISC). RISC recognises and cleaves the corresponding mRNA fragments. Detached mRNAs are subsequently disassembled by cellular nucleases [183]. Therefore, a precise mechanism could be implemented to directly silence the expression of Cx43. Nevertheless, due to the large size and negative charge of siRNAs, which impedes permeability, delivery is one of the main challenges in designing siRNA therapeutics. The use of liposomes, polymers, electroporation, and viral vectors have been previously proposed. In the case of mammals, nasal instillation, local injection, and oral delivery have been described [184].

Ichkova et al. have used siRNA-targeting Cx43 in a rat model with induced juvenile traumatic brain injury. SiRNA was delivered through local injection at the site of injury. The application of siRNA was associated with decreased expression of Cx43 and enhanced motor function recovery [185]. Intriguingly, Cx43-GJ are permeable to siRNA, and this feature has been implemented to develop Cx43-integrated exosomes as nanocarriers able to transport siRNAs [186,187]. Several siRNA-based therapeutic agents have already been developed and approved by the Food and Drug Administration, including patisiran, givosiran, and lumasiran, which were designed to treat hereditary transthyretin-mediated amyloidosis, acute hepatic porphyria, and primary hyperoxaluria type 1, respectively [188].

MiRNAs are short, non-coding molecules composed of approximately 20 nucleotides which take part in the post-transcriptional modification of selected genes. MiRNAs bind to the 3′-untranslated region of their target mRNA and promote its degradation or suppress translation [189]. Downregulated and upregulated miRNAs associated with Cx43 can be implemented in diagnosis, while the application of miRNAs targeting Cx43 could find use in the treatment of atherosclerosis. MiR-144-3p [23], miR-206 [190], and miR-106-a [191] are some of the miRNAs targeting Cx43.

Furthermore, several already known treatment agents and plant derivatives have been found to inhibit Cx proteins as well. Doxorubicin, an anticancer agent, induces Ser368 phosphorylation and reduces Cx43 expression [192]. It has been previously demonstrated that the phosphorylation of Ser368 decreases the permeability of Cx43 [193]. Nevertheless, due to organ toxicity and cell cycle impairment induced by doxorubicin, it is unlikely that it will become a therapeutic agent for non-neoplastic diseases [194]. Gentamicin, an aminoglycoside antibiotic, was also found to disrupt Cx43-GJs and induce the translocation of Cx43 from the cell membrane to the cytoplasm [195]. Furthermore, Subedi et al. showed that synthetically developed kanamycin derivatives are capable of inhibiting Cx43 [196]. Additionally, propofol, a commonly used anaesthetic, and captopril, an ACE inhibitor used to treat hypertension, were both found to suppress Cx43 expression [197,198].

Flavonoids are plant derivatives with antioxidant, anti-inflammatory, and anticoagulant properties [199]. The molecular mechanisms induced by flavonoids include the inhibition of transcription factors associated with inflammation, such as NF-κB and AP1. Moreover, they have been found to suppress cyclooxygenase and ROS production. Consequently, lower levels of pro-inflammatory cytokines are released, such as IL-1β, IL-6, and TNF-α [200]. Baicalein, a *Scutellaria baicalensis* derivative, has been found to reduce the expression of Cx43, P2Y12 purinergic receptors, TNF-α, and IL-1β in rat models [201]. Genistein is another flavonoid found in soy-based products that could inhibit Cx43 expression. Studies showed that genistein downregulates Cx expression in HEK-STING cells, mouse embryonic fibroblasts [202], and HUVECs [203]. Furthermore, Jia et al. demonstrated that genistein suppresses TNF-α-induced expression of ICAM-1 and VCAM-1 in HUVECs, together with subsequent monocyte adhesion. The anti-inflammatory role of genistein was also confirmed in an animal study [204]. Similar effects of genistein were observed in a study by Babu et al. in human aortic EC in hyperglycaemic conditions. Nevertheless, the authors of both studies point to cAMP/protein kinase A as a potential pathway involved in observed results [205]. Nobiletin and pinocembrin were found to inhibit the upregulation or decrease the protein levels of Cx43 and their effect on Cx43 in atherosclerosis-related cells should be examined [206,207].

## 7. Limitations and Future Directions

This review cannot be considered without certain limitations. To begin with, many studies described in this paper examined the role of connexins in cultured cells. These studies carried out experiments on cells directly related to atherosclerosis, such as EC, macrophages, and VSMC, followed by the experiments on animal models. However, some mechanisms depicted in the current review were discovered in other cell types, which makes their correlation with atherosclerosis more speculative. For instance, a potential colocalisation of Cx43 GJ and hemichannels with YAP should be examined in endothelial cells. Furthermore, the precise mechanism of Cx43 enhancement following ox-LDL stimulation should be investigated. Nevertheless, we strongly believe that the described hypotheses might guide future research. Secondly, ATP release through Cx has been one of the major schemes described in this review. However, several other channels are responsible for the ATP release. Apart from connexins, other proteins include pannexins, maxi-anion channels, calcium homeostasis modulator 1, and volume regulated anion channels [208]. Nevertheless, recent studies also search for the potential role of ATP leak through connexins in various diseases, such as renal fibrosis [209] or ischemia-reperfusion injury [210]. Recent difficulties in differentiating the precise roles of pannexin and Cx channels in ATP release result from the non-selective activity of common Cx inhibitors, such as carbenoxolone. Dye uptake, luciferase bioluminescence, and evaluation of the electrophysiological properties are methods which have been used to study these channels [211]. In addition, despite structural similarities, Cx isoforms seem to have a different role in the development of atherosclerosis. The opposite roles might result from the isoform-specific permeability profile [97], or the interaction with other proteins (e.g., Cx40/CD73 or KLF2/Cx37) or between connexin isoforms (Cx37/Cx40). Furthermore, another modulatory mechanism involves the phosphorylation of Cx43 at several sites, which modulates hemichannel permeability and GJ assembly [212]. However, this issue should be further examined. Moreover, Cx gene polymorphisms may play a role in the susceptibility to atherosclerosis, myocardial infarction, and hypertension [213,214,215].

## 8. Conclusions

This review summarises the roles of Cx in the development of atherosclerosis and points to potential therapeutic agents targeting Cx that could be used in the future to treat atherosclerosis. The disease is characterised by pro-inflammatory conditions that stimulate EC, platelets, macrophages, and VSMC to form atherosclerotic plaques. The current evidence suggests that Cx43 contributes to pro-inflammatory conditions and has proatherogenic properties. Alterations in Cx43 expression have been associated with various molecular mechanisms, including YAP translocation, NF-κB activation, ATP release, the stimulation of purinergic receptors, and NLRP3 assembly. Consequently, Cx43 takes part in several processes correlated with the progression of atherosclerosis, such as endothelial dysfunction, monocyte recruitment, macrophage polarisation, VSMC migration, and the inhibition of autophagy. Sequences of Cx43 could be implemented to inhibit its function by developing mimetic peptides. Furthermore, siRNAs, miRNAs, and various already known pharmaceuticals such as propofol or gentamicin can inhibit the function of Cx43. Moreover, phosphorylation of Ser255 and Ser368 represent strategies that could be applied in developing novel agents targeting Cx43. On the contrary, Cx37 and Cx40 are suggested to play a protective role in atherosclerosis by inhibiting NF-κB or suppressing monocyte recruitment. Nevertheless, the precise role of these molecules might depend on the cellular context, as Cx37 has also been linked to the promotion of atherosclerosis [216]. Furthermore, the presence of Cx45 was found in VSMC as well, but its function remains unclear [217]. Despite previous studies, the role of connexins in the pathogenesis of atherosclerosis has not been fully explained. Understanding the involvement of connexins in the pathogenesis of atherosclerosis requires further research, especially clinical studies.

## Figures and Tables

**Table 1:** Summary of the different roles of Cx isoforms in the promotion or suppression of atherosclerosis.

	Role of Hemichannels	Role of Gap Junctions	Other	References
Endothelium	Inhibition of Cx43 hemichannels might reduce disturbed flow induced activation of endothelium. ATP release through hemichannels might contribute to the NLRP3 assembly.	Inhibition of GJ increases vascular stiffness. GJ-mediated communication promotes the activity of tissue factor.	Potential interaction with YAP. Interaction between Cx40 and anti-adhesive CD73. Interaction between Cx40 and sTET1 to promote endothelial barrier enhancement. Interactions between Cx37 and Cx40 and eNOS activity. Expression of Cx43 may be correlated with enhancement of adhesion molecules. Interactions between Cx40 and Cx37 with KLFs to promote quiescent endothelium.	[49,51,57,58,67,94,101,107,108,109,113,115,116,117,118,119].
Platelets	Connexins regulate platelet functions. ATP release through pannexin is correlated with thrombin formation and aggregation.			[121,122,125].
Macrophages	Cx37 hemichannel activity regulates adhesion of macrophages to endothelium. ATP release may promote M1 macrophage polarisation.		Inhibition of Cx43 suppresses monocyte-endothelium adhesion.	[128,129,131,144].
Vascular Smooth Muscle Cells		Cx43 contributes to the VSMC phenotype switch from a contractile into a synthetic state. Cx43-mediated myoendothelial junctions promote a synthetic subtype of SMC.	Cx37 may destabilise Cx43 gap junctions. Cx37 suppresses VSMC proliferation. Cx43 takes part in suppressing VSMC autophagy.	[151,153,164,166].

## Data Availability

Not applicable.
